# East Asian’s Perception of Western Countries’ Urban Hygiene and Public Health in the Late Nineteenth Century: A Review Article

**Published:** 2017-10

**Authors:** You-Ki MIN, Sam-Hun PARK

**Affiliations:** 1. Dept. of History, Kyung Hee University, Seoul, South Korea; 2. Asia Contents Institute, Konkuk University, Seoul, South Korea

**Keywords:** Public health, Urban hygiene, Governmentality, Miasma theory, Sanitization

## Abstract

**Background:**

Modern hygiene administration in Japan and Korea began to be organized in the end of 19th Century by accepting Western public health system. Then, how did the elite in these two East Asian countries recognize and understand Western public health movement in the 19th Century? Answering this question could provide historical knowledge about the background of starting modern hygiene administration in East Asia.

**Methods:**

To understand the birth of modern public health system in East Asia, Japanese and Korean elite’s records on Western countries were reviewed. The documents examined were The Iwakura Embassy 1871–73 published in 1878 as an account of Japanese Meiji government’s special ambassador sent to the United States and Europe, and Seoyugyeonmun (observations on travels in the West) published in 1895 by a Korean intelligent.

**Results:**

The Iwakura Embassy 1871–73 suggested modern water supply and drainage, roadside trees, and parks, to prevent contagious diseases and improve urban hygiene. Seoyugyeonmun emphasizes that hygiene is an important task that civilized government has to be in charge. So, specific tasks of public health should be imposed on sanitary police.

**Conclusion:**

Public health was one of the major factors that contributed to national prosperity in the 19th century. Such recognition enabled organization of hygiene administration to be part of the project pursuing enlightenment, modernization, and civilization at the end of the 19th century in Japan and Korea.

## Introduction

Various political and economic thoughts in Europe in the 18th century showed insistence on the relationship between health and wealth of nations (1). The Modern States needed governmentality, a term invented by Michel Foucault, in order to manage and reproduce healthy population (2). This led to the emergence of Medizinalpolizei (Medical Police) as part of modern administrative systems. The concept of Medizinalpolizei was initially proposed by an Austrian physician, Wolfgang Thomas Rau in 1764 (3), and was first theorized in the literature written by a Prussian physician, Johann Peter Frank in 1779 (4). Johann Peter Frank emphasized the importance of legislation and systemization for public health and maintenance of physician and public health work force according to increase of population (5). Société Royale du Médecine (Royal Society of Medicine) was established in 1772 in France as the first governmental institution in charge of public health issues. It organized a network for reporting local epidemics (6). The French Revolution emphasized the matter of health as one human right. The Constituent Assembly’s Comité de salubrité (Committee on Salubrity) in 1791 asserted the matter of health as one of state responsibilities for citizen (7).

Following mercantilism, Industrial Revolution began in the late 18th century. It was accompanied by rapid urbanization. Concentrated population in industrial cities caused serious housing crisis for the working class. Furthermore, most industrial cities were not equipped with urban infrastructure. They lacked sanitary arrangements and suffered from severe environmental contamination due to emissions and wastewater. In such situation, public opinions on hygiene reform had been gathered, and the urban problems became to be magnified as social questions.

Urban hygiene conditions were investigated and observed by social reformers as well as hygiéniste (hygiene-ideologists). Physician Louis René Villermé published the first issue of *Annales d'hygiène publique et de médecine légale* (*Annals of Public Hygiene and Forensic Medicine*) in France in 1829. He studied effects of socioeconomic status on health based on data of comparative mortality in Paris in the middle of 19th century. He is now recognized as a pioneer in social epidemiology (8). In *Die Lage der Arbeitenden Klasse in England* (*The Condition of the Working Class in England*), German philosopher Friedrich Engels criticized poor hygiene conditions in slums of industrial cities in England (9). English social reformer Sir Edwin Chadwick appointed as a secretary to Poor Law commissioners in 1834 published a report on sanitary condition of the laboring population in 1842 (10). This report contributed to the passing of Public Health Act in 1848 in England, which influenced politicians and social reformers of French Second Republic emerged by 1848 revolution. In France, loi sur l’assainissement des locaux insalubres (Unsanitary Houses Sanitization law) was then established in 1850. Discussion about the law had been actively started since 1849 when cholera was running rampant in France (11).

Fear of cholera in the 19th century contributed to the establishment of legislation for urban hygiene reform in England as well as in France. Cholera was originated from the Indian continent. It was first spread to Russia in 1817 by trade routes. Later it was spread to West Europe, North America and the rest of the world. In 1832, when cholera epidemic first occurred in West Europe, approximately 6000 and 18000 people died of cholera in London and Paris, respectively. In 1849, approximately 15000 and 16000 people died of Cholera in London and Paris, respectively. When deaths continued, resulting in approximately 10000 deaths in London in 1854, physician John Snow characterized cholera as a water-borne epidemic (12).

Experimental Medicine established by French physiologist Claude Bernard in the middle of the 19th century contributed to the improvement of public health. He established scientific method in medicine. Experiment in laboratory was directly associated with the development of microbiology. Louis Pasteur presented his Germ Theory in the meeting of Société Chimique de France (The French Chemical Society) in 1861 (13). Tuberculosis research was conducted in German reported at the causative agent of tuberculosis to be slow-growing *Mycobacterium tuberculosis* in 1882. The innovative growth of microbiology synthesizes Theories of Contagion and Miasma Theory concerning the outbreak of epidemic disease (14). The Germ Theory implies that the spread of epidemic disease is neither the result of direct contact with patient nor that of bad air. Since bacteria can cause epidemic diseases, both personal hygiene and environmental sanitation are now being recognized as important factors for preventing bacteria expansion.

The 1848 Public Health Act in England mentioned earlier regulated the establishment of institution to manage water supply, drainage arrangement, road cleaning, and road pavement under a single administrative system in order to improve urban hygiene conditions. The 1850 loi sur l’assainissement des locaux insalubres (Unsanitary Houses Sanitization law) in France authorized City Council to establish an in-charge committee to investigate hygienic conditions of building and dwelling permit. In London, construction of modern sewers was started in 1856. In Paris, large-scale city renovation was conducted by a prefect of the Seine, Georges-Eugène Haussmann, from 1853 to 1870. It included the demolition of medieval neighborhoods deemed overcrowded and unhealthy, the building of wide avenues, boulevards, new parks and squares, and the construction of new sewers, fountains, and aqueducts (15).

In East Asia, modernized hygienic administration derived from Western countries was accepted at the end of the 19th century. In Japan, modern public health administration was initiated in 1872, starting from the creation of Medical Affairs Board (16). In Korea, the planning of modern public health system was initiated by elites of the Gaehwadang (Enlightenment Party) in the 1880s. It was put into practice between 1894 and 1895 when the overall reform of government organization was carried out.

A number of researchers in Japan and Korea have reviewed initiation of modern hygienic administration. It was found that traditional concepts and knowledge of health in East Asia were converted to modern western concepts and knowledge of health in the early Meiji era (17). In addition, there was a consideration of modern hygienic administration in the end of the 19th century as an origin of modern medical system in Korea (18). Japanese and Korean medical history researchers have published study results regarding interest of East Asian elite in Western and modernized public health policy. However, comparison study on recognition of modern urban sanitation between elites of both countries is still lacking. In studies considering experience and recognition of Western and modernized cities by Iwakura Mission, urban sanitation has been addressed by focusing on the establishment of modern State (19) or characteristics of modern urban civilization (20).

Therefore, the objective of this study was to analyze how Japanese and Korean elites in the end of the 19th century recognized and evaluated Western urban sanitation and public health movement. Results of this study would reveal the logical background of initiating modern administrative system regarding public health and hygiene in East Asia.

## Methods

This study analyzed narratives of two important records of Japanese and Korean elite who traveled to Western countries in the 19th century. Observations and assessments for public health, urban sanitation, medicine-related facilities, systems, institutions, and ideas in these two important kinds of literature were reviewed quantitatively and qualitatively.

The first literature used in this study was *The Iwakura Embassy 1871–73: Account of the Ambassador Extraordinary & Plenipotentiary’s Journey of Observation* published in 5 volumes in 1878. It was an official record combining formal records diplomatic mission sent to the United States and Europe from 1871 to 1873. The leader of this mission was Iwakura Tomomi, noble and statesman. It was written by Japanese Meiji government with additional information after travel (21). This Mission consisted of 107 people, including most major Japanese government officials, several of them became key figures of modern hygienic administration started in Japan after the travel. The second literature used in this study was *Seoyugyeonmun* (Observations on Travels in the West) written by an intelligent Korean, Kil-Chun Yu, from 1887 to 1889. It was published in 1895 (22). Born in 1856 in Seoul, Yu went to Japan in 1881 for a year and studied under Fukuzawa Yukichi, one of the founders of modern Japan. In 1884, Yu traveled to the United States as part of the first official Korean delegation to observe American industry and government. He studied at Massachusetts and came back to Korea in 1885 after traveling several countries in Europe. However, he wrote *Seoyugyeonmun* while he was under house arrest in 1892 due to oppression imposed on modernization advocates. Thereafter, Yu participated in the overall reform of government organization between 1894 and 1895. That became the starting point of modern hygienic administration in Korea.

## Results

*The Iwakura Embassy* comprised five volumes and one hundred chapters. Cities visited and recorded by Iwakura Mission are listed in Table 1.

**Table 1:** List of cities visited and recorded by Iwakura Mission

***Volume***	***Country***	***Year***	***City***
Volume I (Ch.1–20)	The United States of America	1872.1–8	San Francisco, Chicago, Washington D.C., Philadelphia, New York, Boston
Volume II (Ch.21–40)	Britain	1872.8–12	London, Liverpool, Manchester, Glasgow, Edinburgh, Newcastle, Bradford, Sheffield, Birmingham
Volume III (ch.41–60)	Continental Europe 1	1872.12–1873.3	Paris, The Hague, Rotterdam, Leiden, Amsterdam, Berlin
Volume IV (ch.61–81)	Continental Europe 2	1873.4–1873.6	St. Petersburg, Florence, Rome, Naples, Venice, Vienna
Volume V (ch.82–100)	Continental Europe 3, Voyage Home	1873.6–1873.9	Vienna, Berne, Geneva, Lyon, Marseilles, Hong Kong, Shanghai

The Iwakura Mission left many records of water supply and sewers system, avenue/boulevard and parks/gardens to help circulate fresh air, and various types of hospitals in cities of the United States and Europe. The following record of observation on water supply system in Chicago is one of the detailed records related to water supply: “It is the custom in the West to test and examine possible sources of pure water, then bring it through iron pipes from a distance and store it in reservoirs.” Moreover, “after being filtered, it is distributed around the town”. Thus, “the water is sweet, clean and uncontaminated, and all the city inhabitants use it for drinking” (16: v.I, ch.8, p.174). It was rated highly positively as uncontaminated and clean water supply via water analysis and filtration.

In London in the 16th century, “since no method of disposing of sewage was provided, as the population increased, epidemics frequently occurred, which were the cause of much distress” (16: v.II, ch.22, p.41) emphasized the importance of sewe-rage system. The detailed record of observations and positive comment on modern sewerage was presented in the narrative about sewerages in Paris: “sewage pipes protect people’s health by removing the filth from the city” (16: ch.45, p.99). The Iwakura Mission visited a number of industrial cities and left abundant records of various factories and industrial facilities in details. At the same time, they explained that smog and bad air covering sky of industrial cities in dark had a bad influence on public health, resulting in average life expectancy of laborers to be much lower than that of upper and middle classes.

The importance of fresh air was emphasized the most for urban sanitation and public health. The Mission also recorded bad smell in industrial cities. While visiting representative industrial cities in England, they expressed their concern about environmental contamination caused by industrialization and urbanization that resulted in deterioration of public health. The importance of air was also mentioned for cities and towns in European continent in which industrialization came relatively late compared to that in England. Emphasizing the importance of air, Miasma was suggested as a cause of spread of epidemic disease in the records at Berlin as shown in the following: “Air is the single most effective element in preserving our health. It has more power to cure illness than any medicine.” “It is also through air that pestilent miasmas are spread and contagious diseases are transmitted” (16: v.III, ch.58, p.316, p.318).

In short, circulation of fresh air was emphasized for urban hygiene. The Iwakura Mission found that Central Park in New York circulates fresh air in Manhattan, where roads were narrow and houses were built close to each other. Similar observations were recorded about gardens and parks in London and Paris. Parks in cities were evaluated as sources of fresh air, and clean, packaged avenues and boulevards with trees were recognized as enabling air circulation. The boulevards in Paris created because of Haussmannization accordingly imitated tree-lined roads in a number of European cities: the boulevards of new areas in Munich, and the Ringstraße (The Ring Road) built upon tearing down the old rampart in Vienna. The Iwakura Mission also compared the function of road with arteries and veins of human body. “The circulatory system extends throughout the body and maintains good health by carrying nutrition to every part” (16: v.V, ch.90, p.178).

Urban sanitation observed in the United States and Europe was recognized as an essential component for non-Western regions to prosper. The Iwakura Mission described Alexandria, an Egyptian city where they passed through on the way back to Japan, as the following. “In recent years, in keeping with the city’s prosperity, roads have been paved and more trees have been planted. Drainage has been improved so that it is easier to keep the city clean. The city’s development has been rapid”(16: v.V, ch.94, p.267).

*Seoyugyeonmun* consisted of 20 chapters, among which chapter 19 and 20 addressed modern cities in the United States and Europe. Similar to *The Iwakura Embassy,* it suggested that maintenance of cleanness by pavement of road and cleaning, water supply facilities, parks, and gardens all contributed to urban hygiene and esthetics in Western cities. In addition, Paris that was changed to modern city by Haussmannisation was rated higher than London and New York. In Paris, large-scale of urban renovation enabled internal urban zones to be re-arranged.

However, *Seoyugyeonmun* only referred to urban landmarks, major buildings, and famous areas rather than explaining various hygienic facilities in western modern cities in details as described in *The Iwakura Embassy*. Descriptions regarding recognition or evaluation of Western public health not only appears in Chapters 19 and 20, but in the other chapters as well, because the other chapters comprise knowledge related to principles of governmental organization and function, roles of systems and institutions, education, finance, necessities of life, health, industry, and studies in Western society.

In *Seoyugyeonmun*, Yu emphasized health as the right of all citizens. Protecting lives and bodies and being healthy were important for the happiness of human being in chapter 4*,* while relief from hunger and disease was one of six things pursued by enlightened policy in chapter 5. The other five things pursued by enlightened policy were advocacy of freedom, guarantee of freedom of religion, encouragement of technology, academics, and public education, and reliability of the government. Chapter 6 addressed the role of government, focusing on the need and importance of public health and hygienic administration. The government should encourage establishment of parks to help hygiene by providing fresh air to the city. However, the most important role of government in public health was to establish administrative system to prepare and apply hygiene-related rules. It was described in details below.

Hygiene-related rules are one of several significant items the government is involved. Epidemics such as typhus or a mysterious disease always began to spread in non-clean areas with high density of population in metropolitan cities. Diseases such as measles, smallpox, and scarlet fever are caused by human fever. However, they become extremely compromised upon dirty air contact as disease germs are doubled. Epidemics, in fact, are more severe than war situation, as it could become a cruel disaster for human being. In this sense, spread of epidemics could be prevented by observing hygiene-related laws established by government to punish those with severe violation, and by resulting streets and houses to be clean.

Severe application of law to this matter might be taken as ‘too much’ by some people. However, hygiene-related law could prevent disaster. There would be no difference between deaths due to epidemic and those caused by miasma from filth threw onto the street or sewer and death caused by gunshot, bow wounds, or bow. Therefore, hygienic factors should be thoroughly banned by strict application of relevant laws (17: ch.6).

One role of government is to suggest administrative policies, including medical policy for more specific institution for public health administration. Chapter 10 addressed police system. Police were divided into administrative police and judicial police. Functions of administrative police included disaster prevention, public health supervision, corrupted public moral control, and offense against law prevention. Administrative police were in charge of hygiene-related affairs, including epidemic prevention, disinfection and vaccination against smallpox, beverage/food/medication management, and slaughterhouse/cemetery/crematorium management. In chapter 11 of *Seoyugyeonmun, ‘*how to manage health’ was presented in six ways as shown below, all emphasizing the importance of both personal and environmental hygiene for health.

1. It is important for human to enjoy health without suffering from illness, for which the government should take some responsibility.
2. Regular exercising should be made as a habit.
3. Sleeping and eating should be appropriate and regular. It is undesirable to sleep with a number of persons together in a narrow room because exhalation from many people in the narrow room can easily cause illness. Clean clothes should be put on.
4. Maintaining clean house and road is closely related to health maintenance. Typhus or a mysterious disease among epidemics is caused by miasma. Cleaning surroundings of house and road, planting trees around the house, and taking shower often is good ways to remove Miasma.
5. Laws should be observed closely as violation of laws might lead to various kinds of penalties and sufferings that could compromise health.
6. Health policy in a state depends on its government. Therefore, the institution in charge of hygiene should be established by the government and maintained by the town with financial support from tax. The government is responsible for cleaning roads and preventing the spread of epidemics by using disinfection measures.

## Discussion

Major hygienic reform flow in Europe in the middle of 19th century when the importance of public health was increased due to industrialization and urbanization could be summarized as below.

1831–1832 Cholera epidemics in Europe
1842 Edwin Chadwick published his Report on Sanitary Conditions
1848 British Public Health Act
1849 Outbreaks of Cholera in Europe
1850 Unsanitary Houses Sanitization law in France
1853 Compulsory smallpox vaccination in Great Britain
1853–1870 Haussmann’s renovation of Paris to modernize the city
1853–1854 Cholera epidemics in Europe
1854 John Snow proved that cholera was spread through dirty water
1856 Joseph Bazalgette started to build London’s sewers
1857 Start of Vienna Ringstraße (the Ring Road) construction
1861 Louis Pasteur published his Germ Theory
1866 British Public Health Act
1868 British law to improve or demolish slum housing

How much did urban hygiene and public health-related contents provided in the two records of Japanese and Korean elite reflect and represent Western public health and hygiene improvement of the same age? As described earlier, position to accept Miasma Theory was presented several times in both literatures. The importance of Miasma theory obtained by ancient Greek physician Hippocrates and Greco-Roman physician Galen had been emphasized since the 18th century (23). In this theory, diseases were caused by environmental factors such as contaminated water, foul air, and poor hygienic conditions. It significantly influenced the improvement of urban environment in the 19th century. Descriptions regarding the importance of facilities with clean parks, squares, and roads to provide fresh air presented in the two records reflected positive results from evaluation of urban hygiene revolution in Western countries at that time.

British physician John Snow characterized cholera as being spread via dirty water in 1854. Water supplies and sewages observed in a number of cities they visited were described as important hygienic facilities in *The Iwakura Embassy* recorded in the 1870s. Dirty or filth-combined water was described more specifically as a cause of disease in *Seoyugyeonmun* written in 1880s. However, both kinds of literature did not directly provide any knowledge or information about Germ Theory presented by Pasteur in 1861. In fact, the theory presented by Pasteur was a revolutionary change in terms of medical thought. However, there was a period of debate, before the germ theory gained traction, in the late 19th century and early 20th century (24). Great effect of smallpox vaccination was reported in *Seoyugyeonmun*. Vaccination against smallpox was the most successful disease prevention activity in the 18th century. Edward Jenner introduced the first successful vaccine in 1796 (25). Smallpox vaccination was then generalized in many countries during the 19th century. It was accepted by pioneering physicians in East Asia before modern hygienic administration started.

Environmental change in modern cities was what East Asian elite specifically observed and recognized as an important matter of during their travel in Western countries. Haussmannisation of Paris, Ringstraße project of Vienna, and the sewage systems of London were set as models were rated positively in both literatures. *Seoyugyeonmun* written and published later than the Japanese text, include description associated with social hygiene movement emerged in Western countries in the end of the 19th century. The need to prevent alcoholic while regulating pubs/bars with relevant law and the hazard of opium being more severe than epidemics that could harm human and the state was also explained. What was the influence that resulted in the initiation of modern hygienic administration in Japan and Korea then? Direct and indirect relations were confirmed based on a number of historical facts.

Nagayo Sensai born in 1838 came to realize the vital necessity of public health service and sanitation system via his travel to Western countries as a member of Iwakura Mission. He came across the German idea of Hygiene or Gesundheitspflege (health care) during this mission and translated these words into Japanese as eisei (hygiene) when Medical Affairs Board was established in 1872. It was initially under the Ministry of Education. Later it became the Board of Public Health under the Interior Ministry in 1875 and the first director was Nagayo Sensai (17).

In Korea, the planning of public health administration system was initiated by reform-minded elites in the 1880s. It was put into practice between 1894 and 1895 when the overall reform of government organization was carried out during the Gabo Reform. The name Gabo came from the name of the year 1894 in the traditional sexagenary cycle. From 1894 to 1895, Kil-Chun Yu, the author of *Seoyugyeonmun*, worked for the government under Prime Minister Hong-jip Kim who intended to modernize Korea. In 1895, he became Vice Minister of State for the Home Office. The government of Hong-jip Kim established the Board of Health in charge of public health affairs, including epidemic prevention, medication management, and vaccination under the Minister of State for the Home Office.

## Conclusion

*The Iwakura Embassy* and *Seoyugyeonmun,* the most famous travel records on Western countries were published by Japanese and Korean elites, respectively, at the end of the 19th century. They considered public health as a factor for personal happiness as well as national prosperity. Such consideration was suggested more specifically in *Seoyugyeonmun* in which Western systems, institutions, and social organizations were comprehensively explained. However, in *The Iwakura Embassy*, hygienic facilities and specific functions/principles observed in person in visiting cities were described. Nagayo Sensai, one member of the Iwakura Mission, convinced the importance of Western modernized public health system and led the initiation of Japanese modernized public health administration from 1870s to 1880s. Kil-Chun Yu, the author of *Seoyugyeonmun,* highly contributed to the initiation of Korean public health administration in 1890s.

In Western society, industrialization and urbanization accordingly in the 19th century increased interest in environmental factors for public health. The importance of public health and hygiene was confirmed and the need of hygienic revolution was emphasized by East Asian elite during their travel to Western countries. In such historical context, modern public health administration had been commenced since 1870s and 1890s in Japan and Korea, respectively.

## Ethical considerations

Ethical issues (including plagiarism, informed consent, misconduct, data fabrication and/or falsification, double publication and/or submission, and redundancy) have been completely observed by authors.
